# A Short Guide to the Climatic Variables of the Last Glacial Maximum for Biogeographers

**DOI:** 10.1371/journal.pone.0129037

**Published:** 2015-06-11

**Authors:** Sara Varela, Matheus S. Lima-Ribeiro, Levi Carina Terribile

**Affiliations:** 1 Department of Ecology, Faculty of Science, Charles University, Praha, Czech Republic; 2 Museum für Naturkunde. Leibniz Institute for Evolution and Biodiversity Science, Berlin, Germany; 3 Departamento de Ciências Biológicas, Universidade Federal de Goiás–UFG, Jataí, GO, Brazil; University of Porto, PORTUGAL

## Abstract

Ecological niche models are widely used for mapping the distribution of species during the last glacial maximum (LGM). Although the selection of the variables and General Circulation Models (GCMs) used for constructing those maps determine the model predictions, we still lack a discussion about which variables and which GCM should be included in the analysis and why. Here, we analyzed the climatic predictions for the LGM of 9 different GCMs in order to help biogeographers to select their GCMs and climatic layers for mapping the species ranges in the LGM. We 1) map the discrepancies between the climatic predictions of the nine GCMs available for the LGM, 2) analyze the similarities and differences between the GCMs and group them to help researchers choose the appropriate GCMs for calibrating and projecting their ecological niche models (ENM) during the LGM, and 3) quantify the agreement of the predictions for each bioclimatic variable to help researchers avoid the environmental variables with a poor consensus between models. Our results indicate that, in absolute values, GCMs have a strong disagreement in their temperature predictions for temperate areas, while the uncertainties for the precipitation variables are in the tropics. In spite of the discrepancies between model predictions, temperature variables (BIO1-BIO11) are highly correlated between models. Precipitation variables (BIO12- BIO19) show no correlation between models, and specifically, BIO14 (precipitation of the driest month) and BIO15 (Precipitation Seasonality (Coefficient of Variation)) show the highest level of discrepancy between GCMs. Following our results, we strongly recommend the use of different GCMs for constructing or projecting ENMs, particularly when predicting the distribution of species that inhabit the tropics and the temperate areas of the Northern and Southern Hemispheres, because climatic predictions for those areas vary greatly among GCMs. We also recommend the exclusion of BIO14 and BIO15 from ENMs because those variables show a high level of discrepancy between GCMs. Thus, by excluding them, we decrease the level of uncertainty of our predictions. All the climatic layers produced for this paper are freely available in http://ecoclimate.org/.

## Introduction

The last glacial maximum (LGM) was a period of extreme dry and cold climatic conditions [1]. During the LGM, the ice sheets covered large areas of the Northern Hemisphere and the sea level decreased globally an average of 120 meters, modifying the shapes of the continents and altering the current oceanic barriers [2]. Biologists are interested in understanding the impacts that this glacial period had on current and past biotic and genetic diversity. To do this, ecological niche models [3, 4] have been extensively used for mapping the range shifts of species through time as a consequence of the climatic changes [5, 6]. Questions about past species extinction events [7–10], population genetics [11] and population dynamics [12] have been addressed using this methodology.

In this context, ecological niche models are often calibrated and/or projected on paleoclimates simulated with General Circulation Models (GCMs). In general terms, a GCM is a mathematical representation of the physical processes operating in the atmosphere, ocean, cryosphere and land surface and are widely applied for hindcasting (including the LGM scenario) and forecasting the global climatic responses to variations in planetary parameters through time (e.g., solar constant, greenhouse gas concentration, ice sheet dynamics) [13]. Currently, there is an attempt to summarize the knowledge about the climatic dynamics from GCMs (see IPCC Fifth Assessment Report in [14]). Large research projects such as the Coupled Modelling Intercomparison Project (CMIP5: http://cmip-pcmdi.llnl.gov/cmip5/) and the Paleoclimate Modelling Intercomparison Project (PMIP3: https://pmip3.lsce.ipsl.fr/) aim to standardize and coordinate the climate model experiments involving multiple research groups from around the world [15].

However, in spite of the standardization of the basic conditions for the experiments, each GCM has its particularities. Each model uses a particular set of equations to simulate the climatic dynamics, a particular model of global vegetation and a particular resolution to run the simulations (see Table 1). The differences in the initial setup conditions and in their algorithms have the inevitable outcome of ending in different climatic predictions for similarly forced models [15].

**Table 1:** Details of the nine general circulation models (GCMs) used in this comparative study.

Model ID	Modeling Center	Resolution	# of years	Source	Release
CCSM4	National Center for Atmospheric Research, USA	0.9° × 1.25°	100	CMIP5/PMIP3	2012
CNRM-CM5	Centre National de Recherches Meteorologiques / Centre Europeen de Recherche et Formation Avancees en Calcul Scientifique, France	1.4° x 1.4°	200	CMIP5/PMIP3	2012
COSMOS-ASO (FUB)	Freie Universität Berlin, Germany	3.75° x 3.7°	600	PMIP3	2012
GISS-E2-R	NASA Goddard Institute for Space Studies, USA	2.5° x 2.0°	100	CMIP5/PMIP3	2012
FGOALS-g2	National Key Laboratory of Numerical Modeling for Atmospheric Sciences and Geophysical Fluid Dynamics (LASG). Institute of Atmospheric Physics (IAP), China	2.8° × 2.8°	100	CMIP5/PMIP3	2013
IPSL-CM5A-LR	Institut Pierre Simon Laplace, France	3.75° x 1.9°	200	CMIP5/PMIP3	2012
MIROC-ESM	Atmosphere and Ocean Research Institute (University of Tokyo), National Institute for Environmental Studies, and Japan Agency for Marine-Earth Science and Technology, Japan	2.8° × 2.8°	100	CMIP5/PMIP3	2012
MPI-ESM-P	Max Planck Institute for Meteorology, Germany	1.9° x 1.9°	100	CMIP5/PMIP3	2011
MRI-CGCM3	Meteorological Research Institute, Japan	1.1° x 1.1°	100	CMIP5/PMIP3	2012

All simulations were obtained from r1i1p1 ensemble member, except GISS (r1i1p151). The original resolutions of the maps, in decimal degrees (longitude°— latitude), are coarse (between 1° and 4°). Most models were run along a 100 years time-series after the spin-up period. Source: CMIP5, Coupled Model Intercomparison Project Phase 5 (http://cmip-pcmdi.llnl.gov/cmip5/) and PMIP3, Paleoclimate Modelling Intercomparison Project Phase 3 (http://pmip3.lsce.ipsl.fr/).

Although the use of the paleoclimatic simulations to map species distributions during the LGM has increased in recent years, there has been no systematic review to guide researchers to fully understand the uncertainties and particularities of these climatic predictions. In the last years, researchers often used MIROC and/or CCSM models, mainly because they were easily available through the www.worldclim.org web repository (nowadays this changed and it is possible to download more than these two GCMs in worldclim.org). Besides, normally, variable selections are based on species requirements, but not on the inherent uncertainties of those climatic layers.

In this paper, we aim to initiate a debate for biogeographers and macroecologists about the uncertainties of the climatic predictions for the last glacial maximum and how to deal with those uncertainties when working with ecological niche models. We aim to provide a simple guideline to help researchers in selecting the most appropriate GCMs and variables for constructing their maps of species ranges during the LGM. We analyze the predictions of nine GCMs for the LGM scenario (see Table 1) in a three-fold discussion. First, we quantify and map the global spatial patterns of the discrepancies between the GCM predictions. Second, we identify the similarities between models and group them to help researchers choose between GCMs. Third, we analyze the correlations between the predictions of 19 bioclimatic variables across the GCMs to help researchers select the appropriate variables for lessening the uncertainties in their predictions about species ranges during the last glacial maximum.

## Methods

### Raw GCM variables

We used the most recent climatic simulations for the LGM scenario from all coupled atmosphere-ocean general circulation models available in the CMIP5 and PMIP3 databases (Table 1). We downloaded four atmospheric variables per month: precipitation (pr), mean temperature (tas), maximum temperature (tasmax) and minimum temperature (tasmin). All of these are from the same ensemble member (r1i1p1), except GISS (r1i1p151).

### Preparing the layers for comparison

The raw outputs of the GCMs have different spatial resolutions (see Table 1). Thus, in order to compare the climatic predictions of the GCMs, we built a set of climatic layers directly comparable between models (same variables, same geographic extent and same spatial resolution). For doing so, first, we calculate a long-term mean from the time-series data of the GCMs, then we interpolate the obtained values to the same grid, and finally, we constructed the 19 bioclimatic variables based on monthly temperature and precipitation values.

GCMs run long-term simulations for stabilizing paleoclimatic predictions. Thus, we averaged the monthly-predicted values from the entire time span available for each GCM. Most GCMs have 100 years time-series, providing standard long-term means. However, some GCMs stabilized predictions on a longer time-series (see Table 1). For those models, we maintained the original time spans to guarantee reliable long-term means from all GCMs. Temperature variables were transformed from Kelvin to degrees Celsius and precipitation flux (in mm m^-2^ s^-1^) was converted to total monthly precipitation (mm month^-1^), taking into account a month with 30 days according the specific calendar of 360 days year^-1^. The original netCDF files with the GCM outputs were manipulated using the functions from *ncdf* R-package [16].

Next, the long-term means were downscaled to a resolution of 0.5 degree of latitude and longitude. The downscaling procedure followed the standard change-factor approach [17], as follows: (i) firstly we compute the anomalies (also called climate change trends) between LGM and current climate for each raw variable at original GCM resolution, (ii) secondarily we interpolate the climate change trends (instead of LGM climates directly) and the current climate from each GCM to the standard 0.5° grid, and (iii) thirdly we apply interpolated climate change trends to the interpolated current climates to obtain the interpolated layers for LGM. In the first step, the climate change trends for temperature variables were computed as the simple difference between LGM and modern conditions (a standard climate anomaly) from each GCM. For precipitation, climate change trends were computed as ratios of such anomaly in relation to its correspondent modern condition [(LGM—modern)/modern]. We used ratios because they are a robust method in order to maintain the original spatial patterns when accounting for large values, like precipitation [17].

In the second step, we used ordinary kriging technique to statistically downscale raw climate change trends (differences and proportions) and current climate from each GCM to a standard global 0.5° grid. We automated all that interpolation procedure by coupling different function from *gstat* R-package [18] as follow. Interpolations were based on function krige from the 12 nearest observations to a given focal point (instead of fitting an inverse distance weighted power from global neighborhood) and a variogram model. To model the spatial structure in data, a variogram was fitted using the function fit.variogram. This function fits ranges and sills from a variogram model (in our case, a spherical variogram) to a sample variogram. The spherical variogram model was used because it shows a progressive decrease of spatial autocorrelation until some distance, beyond which autocorrelation is zero, like the common spatial structures observed in climate data. From an exponential model, for example, autocorrelation would disappear completely only at an infinite distance, differing from the often observed. The sample variogram was obtained using the function variogram, following the direction with the largest range (i.e. the omnidirectional model type) in each variable and assuming a constant trend for variables (i.e. we did not specify predictor variables to fit sample variogram).

A variety of statistical methods have been used for downscaling spatial data and generating interpolated climate surfaces. We used ordinary kriging technique because it is known to produce reliable continuous interpolated surfaces by considering the spatial structure in raw variables to minimize the variance of the errors [19]. This is an important advantage in relation to other simple linear interpolation techniques (e.g. regression methods, see [19] for an overview), as it is desirable to interpolate climatic simulations which reflect the spatial structure of their boundary conditions (e.g. ice sheet, topography, vegetation, insolation). Moreover, because our dataset is based on climatic simulations, it makes no conceptual sense accounting for effects on observed climate patterns, like coastal influence, terrain barriers, temperature inversions (explicitly accounted by PRISM method, for example; see [20], nor linking weather stations along isoclines from irregularly spaced data points (which, for example, would be obtained by thin-plate spline-fitting techniques like ANUSPLIN; see [21] and [22])).

To test the accuracy of our layers we spatially interpolated temperature and precipitation layers from GCM CCSM4 using other four often used methods (thin-plate splines, inverse distance weighting, trend surface with 12^th^ polynomial regression, natural neighbour), which were highly correlated with our correspondent originally interpolated layers (r > 0.96 for precipitation, except from trend surface method, and r > 0.98 for temperature; S1 Table). Moreover, we evaluated the efficiency of all methods by comparing the values of 5000 spatially correspondent points (~10% of original CCSM4 grid cells) from interpolated (X) and original (Z) layers using mean square errors [MSE = 1/n*Σ(X*i*–Z*i*)^2^]. The points were randomly sampled across geographical space and the general procedure was repeated 1000 times. Kriging method showed the lowest MSEs (S1 Fig). Summarizing, our sensitivity analyses showed that although all methods interpolate climatic layers with similar spatial patterns (high correlations), kriging was the most precise for interpolating both temperature and precipitation (lowest MSE).

Finally, we applied the interpolated climate change trends (differences and ratios) to their correspondent interpolated modern conditions to obtain the interpolated LGM conditions. Working with interpolating anomalies (climate change trends) instead of directly interpolating the original variables from all climate scenarios (e.g. modern and LGM) ensures coherency of the simulated climate patterns across time (see [23] for a similar procedure to guarantee topography coherency across time when using observed modern climates instead of pre-industrial simulations as a control data).

We then used the downscaled layers of the four initial variables of the 12 months—precipitation, mean temperature, maximum temperature and minimum temperature—to construct the 19 bioclimatic variables (see www.worldclim.org): BIO1 = Annual Mean Temperature, BIO2 = Mean Diurnal Range (Mean of monthly (max temp—min temp)), BIO3 = Isothermality (BIO2/BIO7) (* 100), BIO4 = Temperature Seasonality (standard deviation *100), BIO5 = Max Temperature of Warmest Month, BIO6 = Min Temperature of Coldest Month, BIO7 = Temperature Annual Range (BIO5-BIO6), BIO8 = Mean Temperature of Wettest Quarter, BIO9 = Mean Temperature of Driest Quarter, BIO10 = Mean Temperature of Warmest Quarter, BIO11 = Mean Temperature of Coldest Quarter, BIO12 = Annual Precipitation, BIO13 = Precipitation of Wettest Month, BIO14 = Precipitation of Driest Month, BIO15 = Precipitation Seasonality (Coefficient of Variation), BIO16 = Precipitation of Wettest Quarter, BIO17 = Precipitation of Driest Quarter, BIO18 = Precipitation of Warmest Quarter, BIO19 = Precipitation of Coldest Quarter. We followed the standard equations from worldClim database to compute bioclimatic variables (see www.worldclim.org/bioclim). However, BIO1 (annual mean temperature) was obtained directly from GCM simulations (raw variable tas) instead of from average of maximum and minimum temperatures. We decided to use these 19 bioclimatic variables because they are the most often used variables by ecologists and biogeographers for training the ecological niche models. We also created a web-repository to share these variables. These downscaled bioclimatic layers are free to use for research and available for download in the Ecoclimate Database (www.ecoclimate.org) and also in Figshare (http://figshare.com/articles/past_GCMs_Sup_material_PLOS_ONE/1418256). e believe that these 0.5° grids would be highly used in macroecological studies.

### Statistical analysis

We constructed our initial array with the 19 variables of the 9 GCMs using the function abind, from the *abind* package in R [24]. We then chose to run simple statistical analyses. We used the standard deviation between all models to calculate the heterogeneity of the predictions for every pixel using the function apply, from the *base* package in R [25]. For plotting the maps we used *raster* [26] and *maptools* [27], and the coastline shapefile map from Natural Earth (http://www.naturalearthdata.com/downloads/110m-physical-vectors/). For quantifying the agreement between models at each cell we calculated the standard deviation of the 9 GCMs, plus the quartile coefficient of deviation (q3-q1)/q3+q1, in order to have a relative value of the climatic uncertainties. We classified the models into groups by using the correlation between their climatic predictions. We used the function hcluster from the R-library *amap* [28] for running a hierarchical clustering analysis to order the similarities between the model predictions (based on the correlations between the predictions for the same variables), and we set the maximum number of clusters to 4. All R-scripts are available in github/SaraVarela/LGM_climate.

## Results

**Fig 1:**
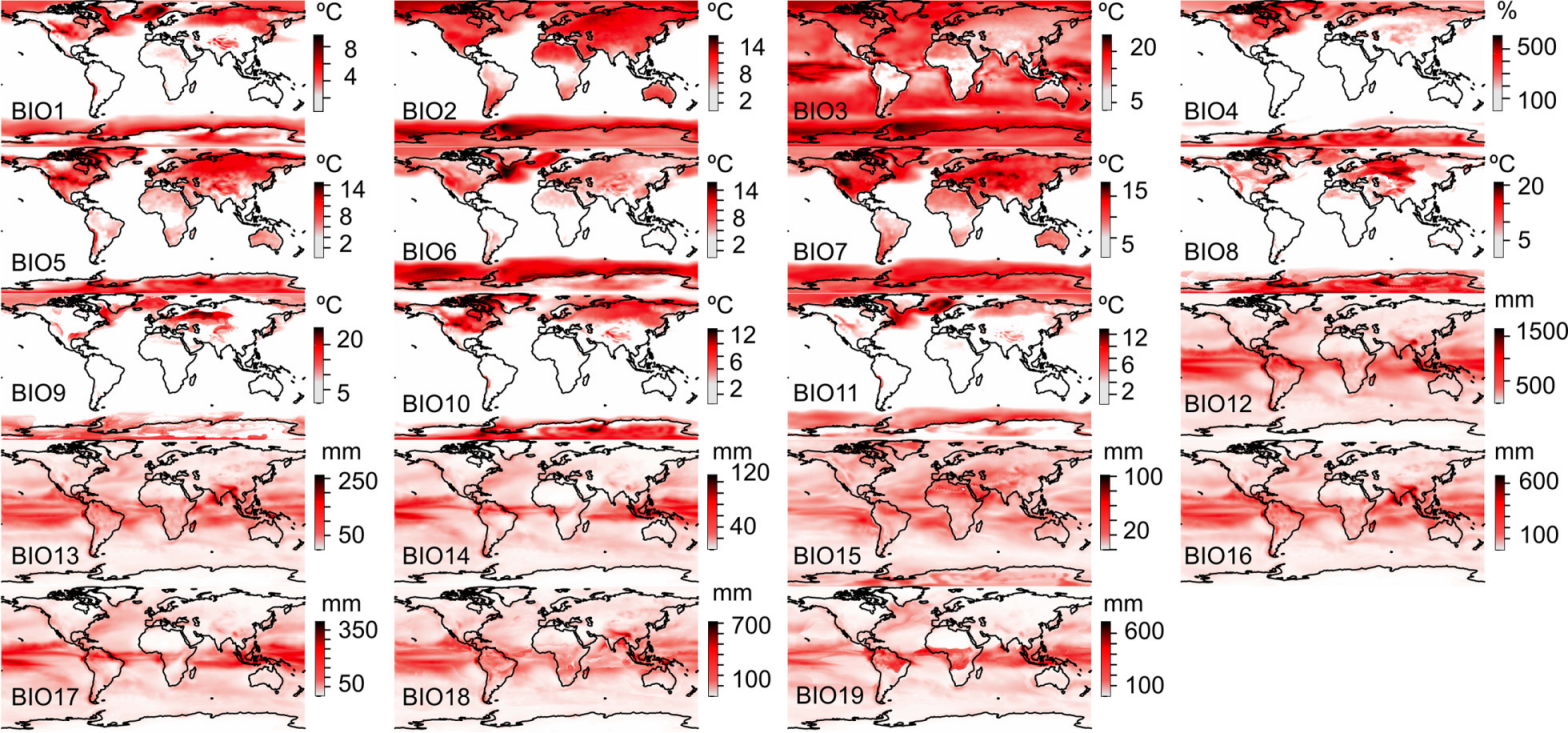
Standard deviation of the climatic conditions predicted by the GCMs. Standard deviation of the temperature variables (BIO1-BIO11) and the precipitation variables (BIO12-BIO19) predicted by the nine General Circulation Models for the last glacial maximum. In general, temperature predictions are more robust for the oceans than for the continents, while precipitation errors are distributed in both seas and continents. Temperature predictions for the last glacial maximum are highly heterogeneous for cold climates, including the mountains, while predictions for tropical, warm and desert environments are more similar between models. On the other hand, tropical areas have highly heterogeneous predictions about precipitation, while predictions for the temperate and cold environments show better agreement.

**Fig 2:**
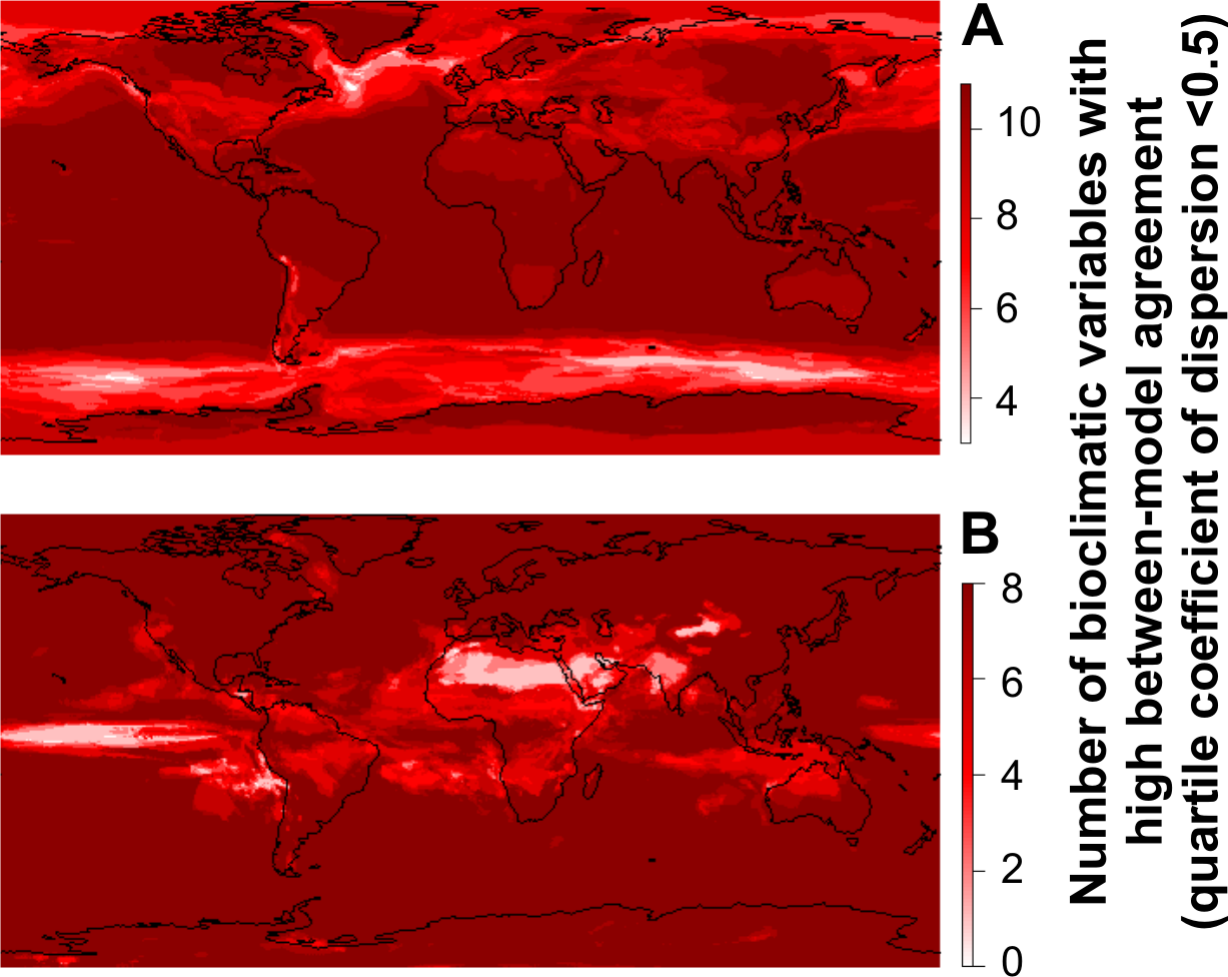
Maps identifying the areas where the bioclimatic predictions for the last glacial maximum show between-model agreement (in dark red) separated by (A) temperature layers (BIO1 to BIO11) and (B) precipitation layers (BIO12-BIO19). Pink areas are those with more climatic uncertainty (GCMs predict different values for temperature and precipitation). These maps are based on the quartile coefficient of dispersion (see methods), which takes into account the dispersion of the predictions related to the actual range of the predictions.

**Fig 3:**
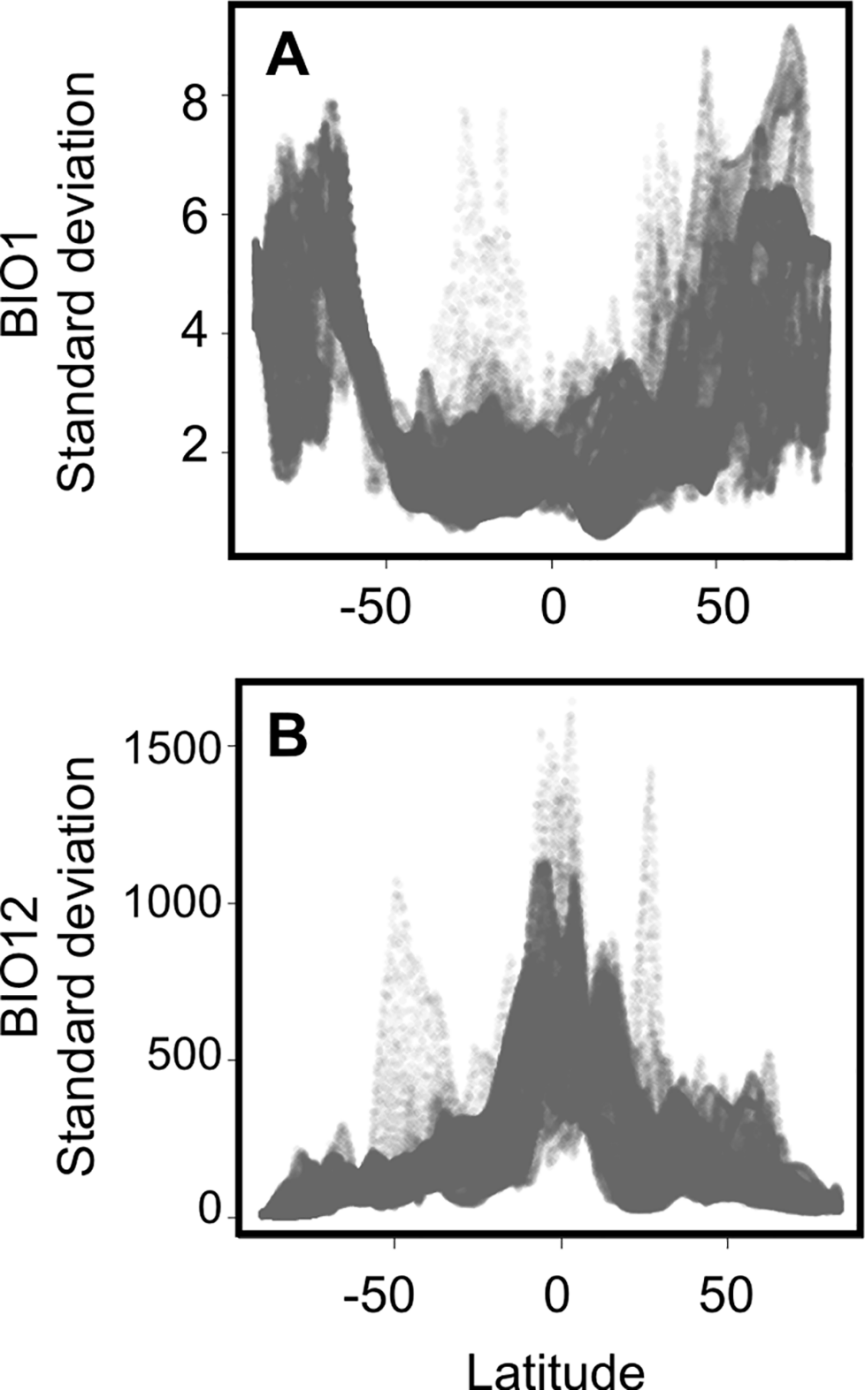
Standard deviation of BIO1 (annual mean temperature) (A) and BIO12 (annual precipitation) (B) in relation to latitude. Our analysis indicates that these variables show an opposite latitudinal distribution of their uncertainties. Temperature predictions diverge at high latitudes, while precipitation predictions have high standard deviations in the tropics.

Our results show that the differences between the GCM predictions are not randomly distributed in geographic space (Figs 1 and 2). Predictions about temperature (BIO1-BIO11) during the LGM have a low deviance in the tropics and a very high deviance in the temperate areas (Figs 1 and 3). Conversely, differences in precipitation (BIO12-BIO19) are located in the tropics (Figs 1 and 3). Comparing the agreement of the model predictions for BIO1 (annual mean temperature) and BIO12 (annual precipitation) across 8 ecoregions we show that Paleartic, Neartic and Antartic ecoregions show low agreements between GCMs for BIO1, while Afrotropical, Indo-Malay, Neotropic, Australia and Oceania show higher levels of agreement between models (S2 Fig). Precipitation values show the exact opposite pattern (S3 Fig).

**Fig 4:**
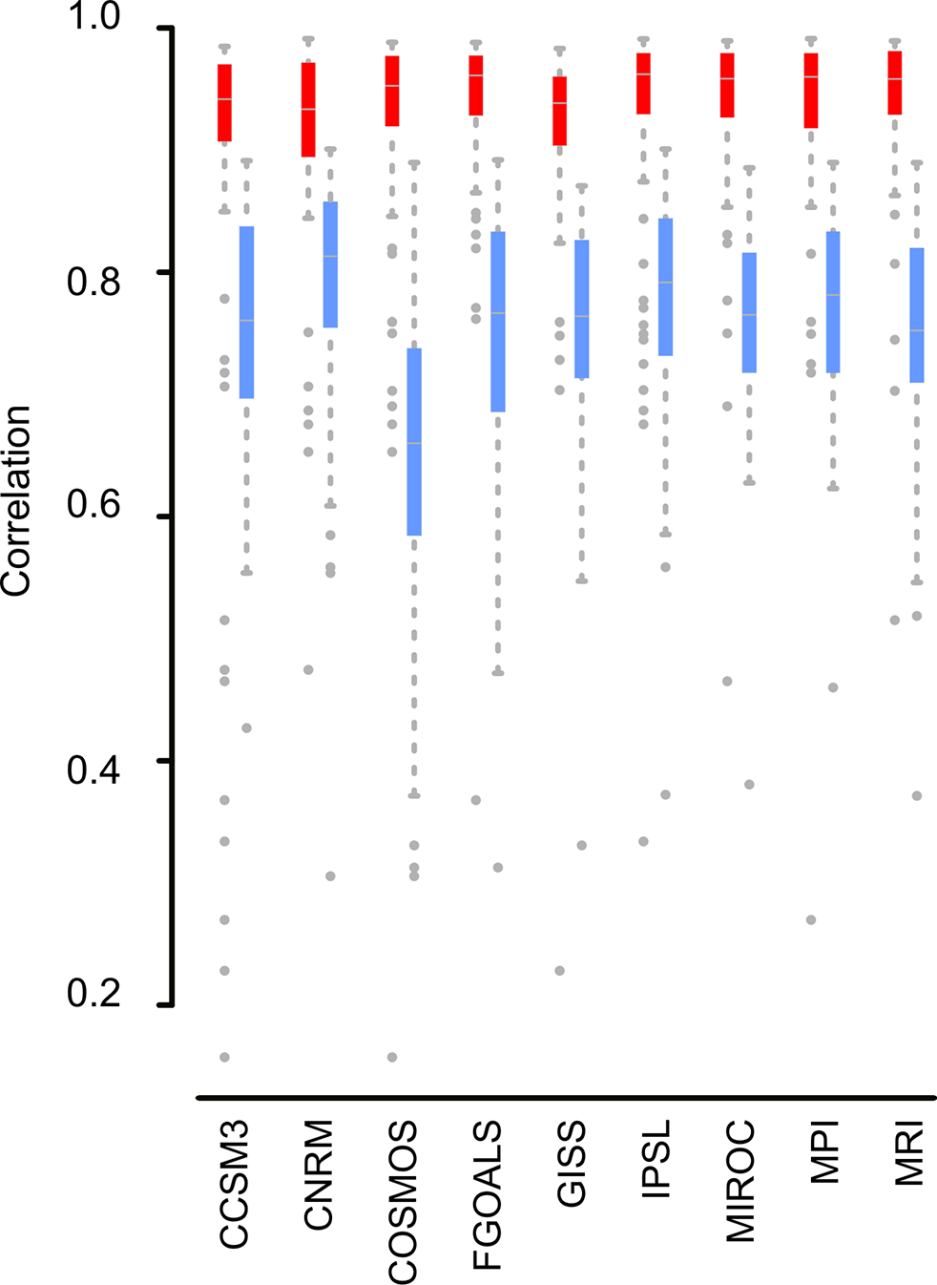
Boxplot showing the correlation values for the temperature variables (in red) and the precipitation variables (in blue), of each General Circulation Model (GCM) compared with the rest of the layers from the other GCMs. Although there are discrepancies in certain variables, temperature variables are highly congruent between models. On the other hand, precipitation variables show more discrepancies between models. COSMOS is the most different model in relation to its predictions about precipitation. Points are outliers (located 1.5 times the interquartile range above the upper quartile and bellow the lower quartile, which is the default definition of outlier in the R function *boxplot*).

After calculating the correlations between the model’s predictions for each climatic layer, we observed that temperature variables are highly correlated; some models have more extreme temperature predictions for the temperate and cold areas, but all the temperature predictions are highly correlated. Precipitation variables show more discrepancies between models; correlations between the predicted precipitation layers between models are not high, and the COSMOS climatic model showed the most different predictions (Fig 4 and S2 Table).

**Fig 5:**
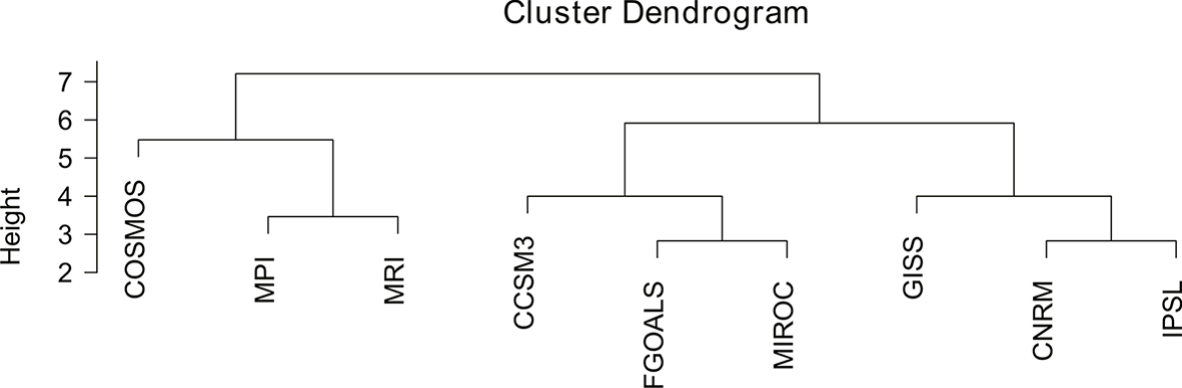
Hierarchical cluster grouping the nine GCMs by the correlation of their predictions for all 19 bioclimatic variables.

By identifying the groups of models with similar predictions, our hierarchical clustering analysis suggests that the four GCMs that should be selected to cover the widest range of climatic predictions for the LGM are: COSMOS (the model with the most singular predictions); one model from the group MPI and MRI; one model from the group CCSM3, FGOALS and MIROC; and the last model from the group GISS, CNRM and IPSL (Fig 5 and Table 2). However, the selected GCMs are dependent on the variables that we choose to use in our analysis. For instance, if we only consider BIO1 and BIO12 (Mean Annual Temperature and Annual Precipitation), the groups of GCMs that cover all the variability between models for these two variables are different. In this case, the simplest combination to cover all the variability between GCM predictions for the LGM would be the group CCSM3, GISS, MRI, and one model from the group of COSMOS and MPI (Table 2).

**Table 2:** Results from the hierarchical clustering analysis (k = 4) identifying the groups of general circulation models (GCMs) that have similar predictions for each variable at a global scale.

	BIO1	BIO2	BIO3	BIO4	BIO5	BIO6	BIO7	BIO8	BIO9	BIO10	BIO11	BIO12	BIO13	BIO14	BIO15	BIO16	BIO17	BIO18	BIO19
CCSM3	1	1	1	1	1	1	1	1	1	1	1	1	1	1	1	1	1	1	1
CNRM	2	2	2	2	2	2	2	2	2	2	2	2	1	2	2	1	2	2	2
COSMOS	2	1	3	3	3	3	3	1	1	3	3	3	2	3	3	2	3	3	3
FGOALS	3	3	2	3	3	2	4	1	3	1	2	4	3	2	4	3	4	1	1
GISS	4	4	2	4	4	4	4	3	3	4	4	4	3	2	4	3	4	1	1
IPSL	2	1	4	3	3	2	4	4	3	3	2	2	1	2	1	1	2	1	2
MIROC	3	3	2	2	3	2	2	1	1	1	2	1	1	4	1	1	1	4	4
MPI	2	4	2	3	3	2	4	4	3	3	2	3	2	4	1	2	3	3	3
MRI	3	3	2	3	3	2	4	4	4	3	2	2	4	2	1	4	2	2	2

Groups have been constructed using the correlation coefficients between layers. Models in the same group indicate that their predictions are highly correlated. This table may help researchers to select the GCMs for constructing their ecological niche models, to generate a more complete picture of the potential variability of the species ranges. Researchers should choose at least one model from each different group for each variable used for constructing their ecological niche models.

**Fig 6:**
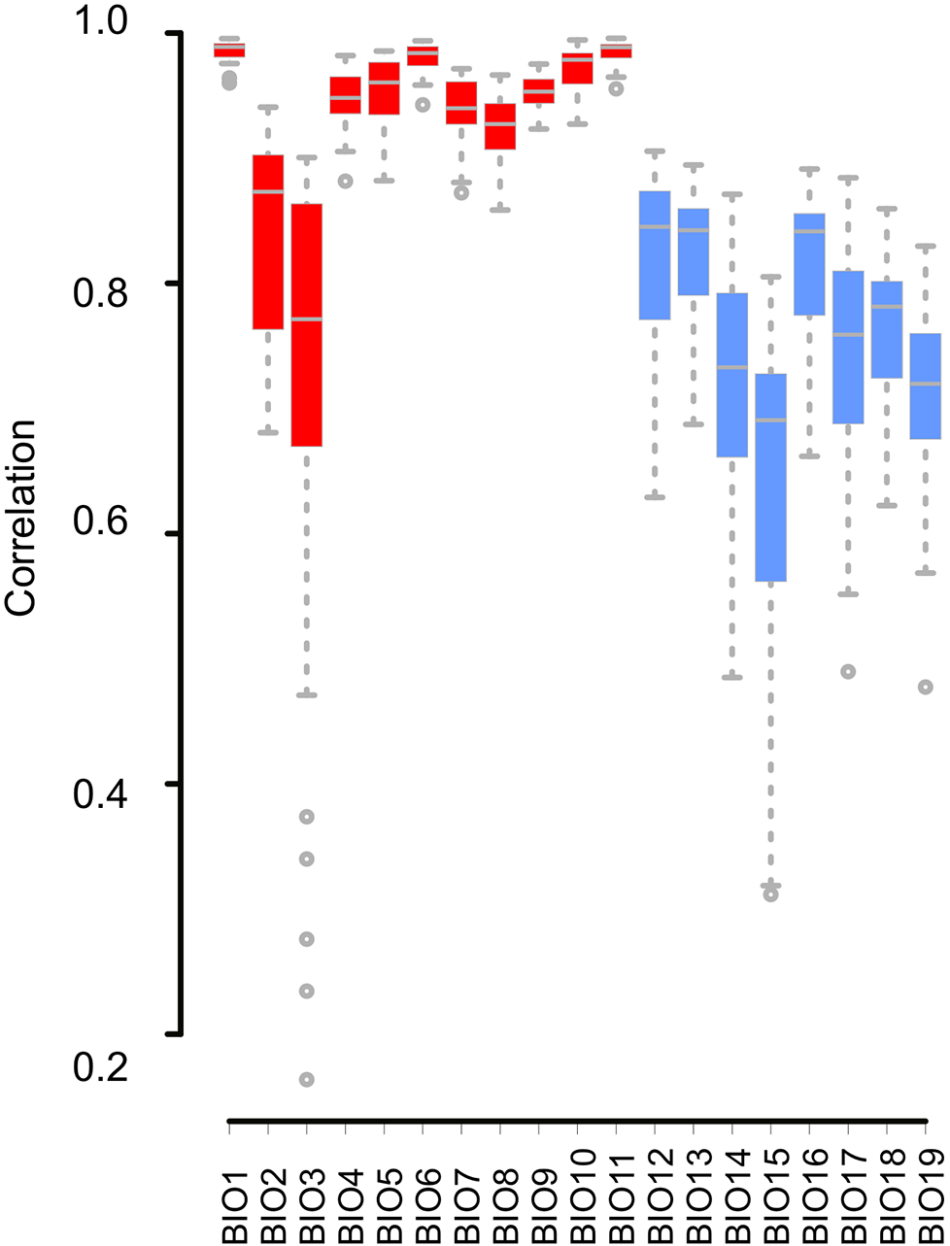
Boxplot showing the correlation between GCM predictions for each bioclimatic variable.

Finally, each temperature variable (BIO1-BIO11) shows high correlations and low deviations between models, with two exceptions: BIO2, Mean Diurnal Range (Mean of monthly (max temp—min temp)) and BIO3, Isothermality (BIO2/BIO7) (* 100). Moreover, precipitation variables (BIO12-BIO19) show intermediate correlations, and BIO14 (Precipitation of the driest month) and BIO15 (precipitation seasonality) show the lowest correlations between the model predictions (Fig 6, S2 Table).

## Discussion

**Fig 7:**
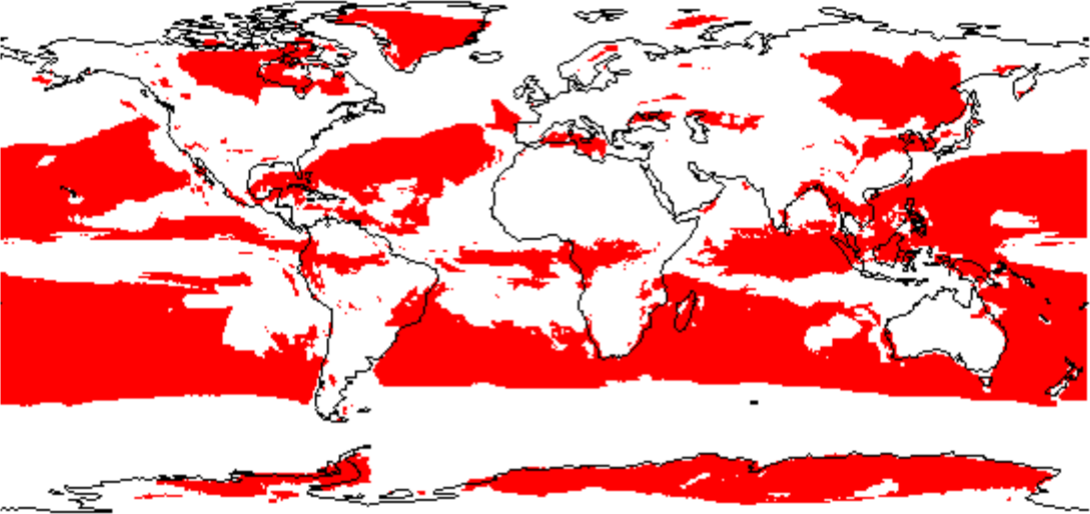
Areas with high agreement between models. Red areas show high agreement across the 19 bioclimatic variables (quartile coefficient of dispersion lower than 0.5 all variables at each cell).

In general, continental climatic predictions show lower agreement between models than oceanic climatic predictions (Fig 7), and some regions in the continents show a high consensus between GCM predictions (such as Greenland and North-East Asia), while others show a low consensus regarding climatic conditions (such as Europe, North Africa and Australia) (see Fig 7). Thus, our analysis suggests that the robustness of the predictions about the distribution of species in the LGM is strongly dependent on the geographic extent of the studies. Until now, the uncertainties about the climatic predictions of the GCMs were not generally discussed in studies about biogeography, but see [29, 30]. The discussions have mainly focused on the biases of the data [31],[32] or the differences between the solutions found by different algorithms for constructing the ecological niche models [33]. However, the climatic predictions from the GCMs do not converge to one unique solution regarding the spatial pattern of the climatic layers in the LGM (Fig 1). We assume that the GCMs will improve their climatic predictions, and in the near future should reach a stronger consensus. Meanwhile, a practical solution for biogeographical studies is to include a wide array of climatic simulations (GCMs) and discuss the potential uncertainties of the so-obtained predictions. We strongly suggest the consideration of different GCMs when predicting the LGM ranges of species suspected to inhabit areas with a low level of consensus between models (Fig 7), in order to cover the observed variability in climatic predictions. However, questions remain. Which models should be used? How many? And further, which variables should we choose in order to decrease the uncertainties of our species range maps?

### Selection of the GCMs

Given our results, we suggest the selection of at least four different GCMs for calibrating and projecting ecological niche models (ENM) in the LGM attempting to cover all the variability observed between the GCMs (see Results and Table 2). However, as discussed above, there is no single solution about which models should be included. We grouped the GCMs according to their similarities across variables (see Table 2), and we show that GCM groups are not identical across the 19 bioclimatic variables. This suggests that, for example, GCMs that have similar predictions for BIO1 and thus belong to the same group, diverge in their predictions for BIO2, BIO3, etc. Therefore, our results suggest that there is no general solution about which GCMs should be included in our studies about the past ranges of species to cover all the variability of climatic predictions. This is dependent on the variables used for constructing our ENM and on the extent of our analysis.

Averaging the predictions of different GCMs is a common procedure in biogeography. However, doing an ensemble of different GCMs is based on the idea that the errors are normally distributed between the GCM predictions and that the mean of those maps will be a close proxy of the “truth”. Also, averaging the species range predictions smoothes the species geographic shifts, hiding extreme optimistic or pessimistic predictions. For all these reasons, we recommend to repeat the models (the model calibration and the map projections) with different GCMs in order to have different measures of range shift, area, etc. according to the different GCMs. And, after that, show the dispersion of the obtained results. This is a more conservative approach that allows researchers to include the GCMs uncertainties in their results. Table 2 should help researchers select the adequate GCMs for their models considering the selected variables at a global scale. Continental and regional scales will be analyzed in the future.

### Selection of the bioclimatic variables

A priori, the selection of the climatic layers used to calibrate ecological niche models could be based on the climatic requirements of the focal species. However, here we deal with this issue from a different perspective; we investigate the correlations between the predictions of each bioclimatic layer across the different GCMs (Fig 6). Our results show that in general, ENMs calibrated with temperature layers will be more robust than ENMs calibrated using precipitation layers, because temperature layers show more agreement between GCMs (Fig 6). These analyses support the results observed with real data; some former versions of the GCMs failed to predict precipitation conditions estimated using pollen records [34].

Between the precipitation layers, two variables, BIO14 (Precipitation of Driest Month) and BIO15 (Precipitation Seasonality), show a high variability between GCMs (Fig 6). Consequently, if we need to include precipitation variables, excluding certain variables (like BIO14 and BIO15) would increase the robustness of the ENM's predictions.

Further, if we want to use both temperature and precipitation layers, not including BIO2 (Mean Diurnal Range (Mean of monthly (max temp—min temp)), BIO3 (Isothermality), BIO 14 and BIO15, for calibrating or projecting the ENMs on the LGM scenario would highly decrease the uncertainties of our species range predictions.

This method for discarding climatic variables does not take into consideration the biotic requirements of the species, but rather the intrinsic uncertainties of the climatic models for the LGM. In macroecological studies that deal with a large number of species at a continental level, applying these types of criteria for discarding problematic variables will increase the robustness of our map predictions. However, if working with a particular species that is highly sensitive to those variables, e.g. to the precipitation of the driest month (BIO14), then we suggest projecting the species niche using all the climatic scenarios from the nine GCMs, to obtain a more complete picture of the solutions according to the different GCMs.

To summarize, climatic predictions for the last glacial maximum show different levels of agreement across space, and, in general, temperature layers show higher consensus than precipitation layers. Thus, when mapping the distribution of species in the last glacial maximum we should try to 1) select different GCMs for constructing our ENM and show the results of our predictions with their associated uncertainty; 2) if possible, exclude the variables that show high levels of uncertainty in our study area (normally precipitation variables) in order to reduce the differences between our predictions.

## Supporting Information

S1 Fig Comparison among interpolation techniques for temperature and precipitation. Comparison among interpolation techniques for temperature (A) and precipitation (B) layers. Boxplots show the mean square errors [MSE = 1/n*Σ(X*i*–Z*i*)^2^] from interpolated (X) and original (Z) climatic values.
(DOC)

S2 Fig Differences between models: Annual Mean Temperature. Standard deviation of the predictions of the 9 different General Circulation Models (GCMs) for the last glacial maximum annual mean temperature (Bio1) across the 8 WWF ecoregions (ecoregions downloaded from http://maps.tnc.org/files/metadata/TerrEcos.xml). Paleartic, Neartic and Antartic ecoregions are the ones with higher differences between GCMs, which means a less agreement between climatic predictions for Bio1 (Annual Mean Temperature). On the other hand, Afrotropical, Indo-Malay, Neotropic, Australia and Oceania show higher levels of agreement between models.
(DOC)

S3 Fig Differences between models: Annual Precipitation. Standard deviation of the predictions of the 9 different GCMs for the last glacial maximum annual precipitation (Bio12). In this case, Antartic, Neartic and Paleartic show higher agreement between GCMs than Afrotropic, Indo-Malay, Neotropic and Oceania regions.
(DOC)

S1 Table Comparative of different interpolation techniques. Correlation (Pearson's coefficient—r) among interpolated temperature (above diagonal) and precipitation (below diagonal) layers from distinct techniques. Krige: ordinary kriging, IDW: inverse distance weighting, Splines: thin-plate spline; Trend: trend surface with 12^th^ polynomial regression; NN: natural neighbor.
(DOC)

S2 Table Correlation coefficients of climatic variables between GCMs. Correlation between the same variables of the different GCMs. Temperature layers (BIO1-11) show better agreement between models than precipitation layers (BIO12-19).
(DOC)
