# Supplementary material for: A Short Guide to the Climatic Variables of the Last Glacial Maximum for Biogeographers
Source: PLoS One. 2015 Jun 11;10(6):e0129037. doi: 10.1371/journal.pone.0129037 (PMC4466021; doi:10.1371/journal.pone.0129037)

**S1 Figure.** **Comparison among interpolation techniques for temperature and precipitation.** Comparison among interpolation techniques for temperature (A) and precipitation (B) layers. Boxplots show the mean square errors [MSE = 1/n*Σ(X*i* – Z*i*)^2^] from interpolated (X) and original (Z) climatic values.


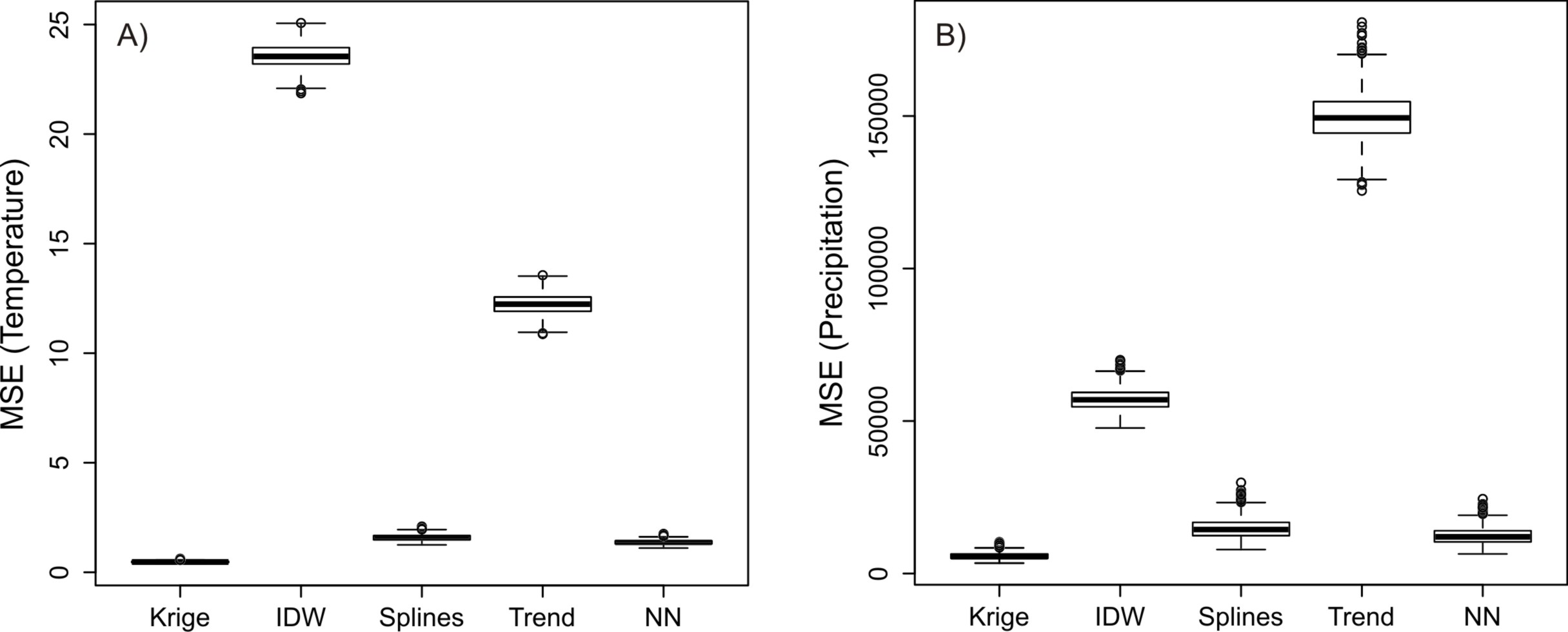

Supplement: S1 Fig — Comparison among interpolation techniques for temperature (A) and precipitation (B) layers. Boxplots show the mean square errors [MSE = 1/n*Σ(Xi–Zi)2] from interpolated (X) and original (Z) climatic values. (DOC) [file pone.0129037.s001.doc]
