# Supplementary material for: A Short Guide to the Climatic Variables of the Last Glacial Maximum for Biogeographers
Source: PLoS One. 2015 Jun 11;10(6):e0129037. doi: 10.1371/journal.pone.0129037 (PMC4466021; doi:10.1371/journal.pone.0129037)

**S2 Figure. Differences between models: Annual Mean Temperature.** Standard deviation of the predictions of the 9 different General Circulation Models (GCMs) for the last glacial maximum annual mean temperature (Bio1) across the 8 WWF ecoregions (ecoregions downloaded from <http://maps.tnc.org/files/metadata/TerrEcos.xml>). Paleartic, Neartic and Antartic ecoregions are the ones with higher differences between GCMs, which means a less agreement between climatic predictions for Bio1 (Annual Mean Temperature). On the other hand, Afrotropical, Indo-Malay, Neotropic, Australia and Oceania show higher levels of agreement between models.


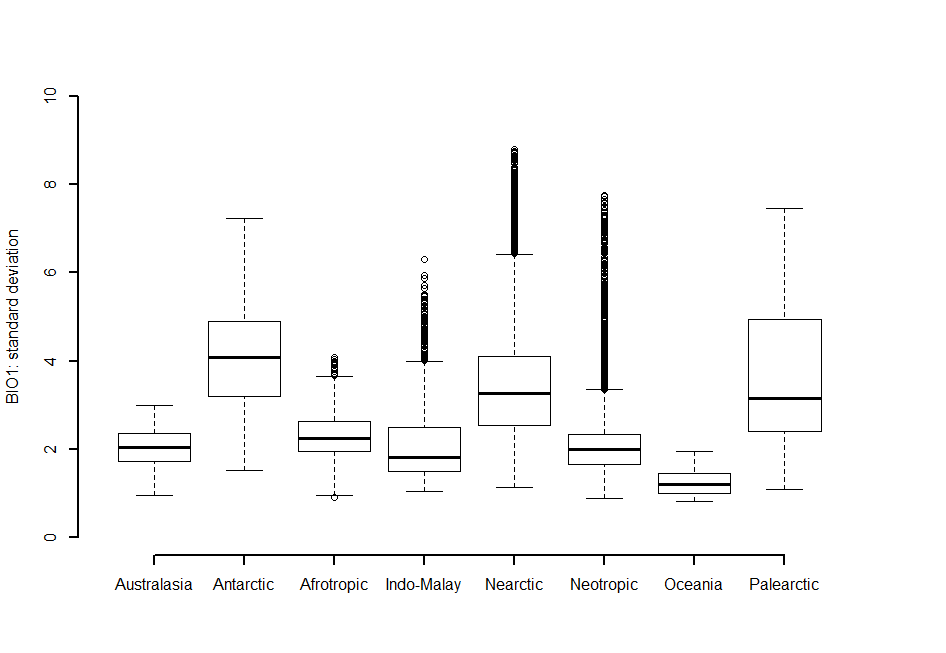

Supplement: S2 Fig — Standard deviation of the predictions of the 9 different General Circulation Models (GCMs) for the last glacial maximum annual mean temperature (Bio1) across the 8 WWF ecoregions (ecoregions downloaded from http://maps.tnc.org/files/metadata/TerrEcos.xml). Paleartic, Neartic and Antartic ecoregions are the ones with higher differences between GCMs, which means a less agreement between climatic predictions for Bio1 (Annual Mean Temperature). On the other hand, Afrotropical, Indo-Malay, Neotropic, Australia and Oceania show higher levels of agreement between models. (DOC) [file pone.0129037.s002.doc]
