# Supplementary material for: A Short Guide to the Climatic Variables of the Last Glacial Maximum for Biogeographers
Source: PLoS One. 2015 Jun 11;10(6):e0129037. doi: 10.1371/journal.pone.0129037 (PMC4466021; doi:10.1371/journal.pone.0129037)

**S3 Figure. Differences between models: Annual Precipitation.** Standard deviation of the predictions of the 9 different GCMs for the last glacial maximum annual precipitation (Bio12). In this case, Antartic, Neartic and Paleartic show higher agreement between GCMs than Afrotropic, Indo-Malay, Neotropic and Oceania regions.


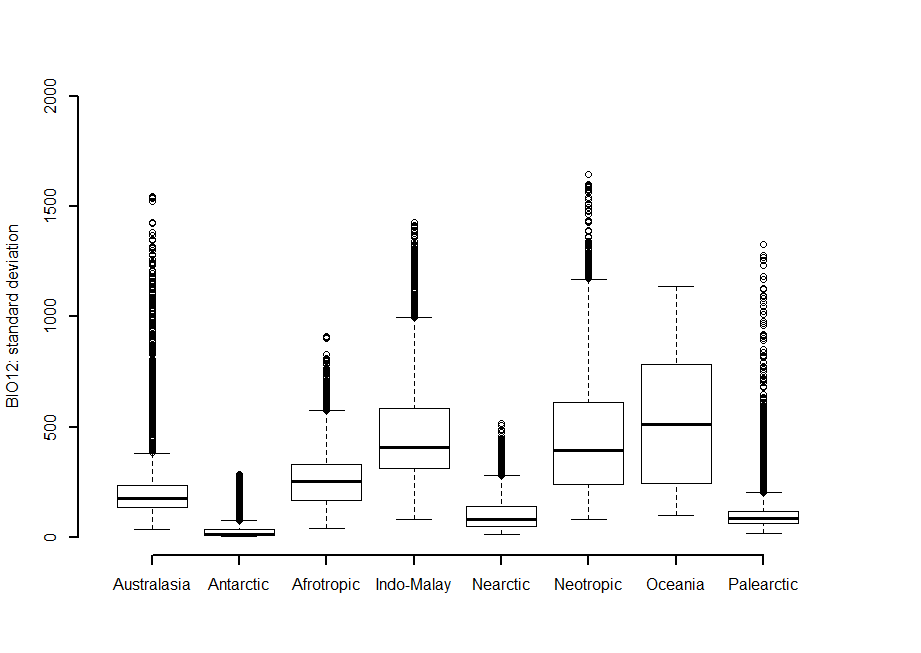

Supplement: S3 Fig — Standard deviation of the predictions of the 9 different GCMs for the last glacial maximum annual precipitation (Bio12). In this case, Antartic, Neartic and Paleartic show higher agreement between GCMs than Afrotropic, Indo-Malay, Neotropic and Oceania regions. (DOC) [file pone.0129037.s003.doc]
