# Supplementary material for: A Short Guide to the Climatic Variables of the Last Glacial Maximum for Biogeographers
Source: PLoS One. 2015 Jun 11;10(6):e0129037. doi: 10.1371/journal.pone.0129037 (PMC4466021; doi:10.1371/journal.pone.0129037)
Supplement: S1 Table — Correlation (Pearson's coefficient—r) among interpolated temperature (above diagonal) and precipitation (below diagonal) layers from distinct techniques. Krige: ordinary kriging, IDW: inverse distance weighting, Splines: thin-plate spline; Trend: trend surface with 12th polynomial regression; NN: natural neighbor. (DOC) [file pone.0129037.s004.doc]

**S1 Table. Comparative of different interpolation techniques.** Correlation (Pearson's coefficient - r) among interpolated temperature (above diagonal) and precipitation (below diagonal) layers from distinct techniques. Krige: ordinary kriging, IDW: inverse distance weighting, Splines: thin-plate spline; Trend: trend surface with 12^th^ polynomial regression; NN: natural neighbor.

|  | Krige | IDW | Splines | Trend | NN |
| --- | --- | --- | --- | --- | --- |
| Krige | - | 0.99 | 0.99 | 0.99 | 0.99 |
| IDW | 0.98 | - | 0.99 | 0.99 | 0.99 |
| Splines | 0.99 | 0.97 | - | 0.99 | 1.00 |
| Trend | 0.84 | 0.90 | 0.83 | - | 0.99 |
| NN | 0.99 | 0.97 | 0.99 | 0.83 | - |
