# Supplementary material for: A Short Guide to the Climatic Variables of the Last Glacial Maximum for Biogeographers
Source: PLoS One. 2015 Jun 11;10(6):e0129037. doi: 10.1371/journal.pone.0129037 (PMC4466021; doi:10.1371/journal.pone.0129037)
Supplement: S2 Table — Correlation between the same variables of the different GCMs. Temperature layers (BIO1-11) show better agreement between models than precipitation layers (BIO12-19). (DOC) [file pone.0129037.s005.doc]

**S2 Table. Correlation coefficients of climatic variables between GCMs.** Correlation between the same variables of the different GCMs. Temperature layers (BIO1-11) show better agreement between models than precipitation layers (BIO12-19)

| **Variable** | **Model** | **CCSM3** | **CNRM** | **COSMOS** | **FGOALS** | **GISS** | **IPSL** | **MIROC** | **MPI** | **MRI** |
| --- | --- | --- | --- | --- | --- | --- | --- | --- | --- | --- |
| **BIO1** | **CCSM3** | NA | 0.982 | 0.988 | 0.980 | 0.960 | 0.978 | 0.987 | 0.983 | 0.989 |
| **BIO1** | **CNRM** | 0.982 | NA | 0.989 | 0.988 | 0.979 | 0.994 | 0.991 | 0.994 | 0.994 |
| **BIO1** | **COSMOS** | 0.988 | 0.989 | NA | 0.982 | 0.964 | 0.989 | 0.992 | 0.992 | 0.990 |
| **BIO1** | **FGOALS** | 0.980 | 0.988 | 0.982 | NA | 0.986 | 0.988 | 0.993 | 0.989 | 0.991 |
| **BIO1** | **GISS** | 0.960 | 0.979 | 0.964 | 0.986 | NA | 0.976 | 0.976 | 0.977 | 0.977 |
| **BIO1** | **IPSL** | 0.978 | 0.994 | 0.989 | 0.988 | 0.976 | NA | 0.991 | 0.996 | 0.991 |
| **BIO1** | **MIROC** | 0.987 | 0.991 | 0.992 | 0.993 | 0.976 | 0.991 | NA | 0.991 | 0.994 |
| **BIO1** | **MPI** | 0.983 | 0.994 | 0.992 | 0.989 | 0.977 | 0.996 | 0.991 | NA | 0.992 |
| **BIO1** | **MRI** | 0.989 | 0.994 | 0.990 | 0.991 | 0.977 | 0.991 | 0.994 | 0.992 | NA |
| **BIO2** | **CCSM4** | NA | 0.712 | 0.910 | 0.854 | 0.733 | 0.890 | 0.783 | 0.723 | 0.868 |
| **BIO2** | **CNRM** | 0.712 | NA | 0.681 | 0.898 | 0.902 | 0.692 | 0.931 | 0.885 | 0.879 |
| **BIO2** | **COSMOS** | 0.910 | 0.681 | NA | 0.824 | 0.753 | 0.906 | 0.755 | 0.764 | 0.851 |
| **BIO2** | **FGOALS** | 0.854 | 0.898 | 0.824 | NA | 0.912 | 0.849 | 0.939 | 0.883 | 0.930 |
| **BIO2** | **GISS** | 0.733 | 0.902 | 0.753 | 0.912 | NA | 0.762 | 0.903 | 0.941 | 0.896 |
| **BIO2** | **IPSL** | 0.890 | 0.692 | 0.906 | 0.849 | 0.762 | NA | 0.781 | 0.730 | 0.812 |
| **BIO2** | **MIROC** | 0.783 | 0.931 | 0.755 | 0.939 | 0.903 | 0.781 | NA | 0.887 | 0.912 |
| **BIO2** | **MPI** | 0.723 | 0.885 | 0.764 | 0.883 | 0.941 | 0.730 | 0.887 | NA | 0.902 |
| **BIO2** | **MRI** | 0.868 | 0.879 | 0.851 | 0.930 | 0.896 | 0.812 | 0.912 | 0.902 | NA |
| **BIO3** | **CCSM5** | NA | 0.480 | 0.164 | 0.374 | 0.234 | 0.340 | 0.471 | 0.276 | 0.521 |
| **BIO3** | **CNRM** | 0.480 | NA | 0.658 | 0.870 | 0.849 | 0.756 | 0.901 | 0.869 | 0.889 |
| **BIO3** | **COSMOS** | 0.164 | 0.658 | NA | 0.767 | 0.764 | 0.681 | 0.695 | 0.820 | 0.708 |
| **BIO3** | **FGOALS** | 0.374 | 0.870 | 0.767 | NA | 0.854 | 0.776 | 0.836 | 0.897 | 0.873 |
| **BIO3** | **GISS** | 0.234 | 0.849 | 0.764 | 0.854 | NA | 0.709 | 0.829 | 0.899 | 0.852 |
| **BIO3** | **IPSL** | 0.340 | 0.756 | 0.681 | 0.776 | 0.709 | NA | 0.782 | 0.755 | 0.750 |
| **BIO3** | **MIROC** | 0.471 | 0.901 | 0.695 | 0.836 | 0.829 | 0.782 | NA | 0.858 | 0.871 |
| **BIO3** | **MPI** | 0.276 | 0.869 | 0.820 | 0.897 | 0.899 | 0.755 | 0.858 | NA | 0.869 |
| **BIO3** | **MRI** | 0.521 | 0.889 | 0.708 | 0.873 | 0.852 | 0.750 | 0.871 | 0.869 | NA |
| **BIO4** | **CCSM6** | NA | 0.918 | 0.937 | 0.947 | 0.882 | 0.946 | 0.952 | 0.936 | 0.943 |
| **BIO4** | **CNRM** | 0.918 | NA | 0.905 | 0.940 | 0.907 | 0.935 | 0.965 | 0.914 | 0.942 |
| **BIO4** | **COSMOS** | 0.937 | 0.905 | NA | 0.954 | 0.920 | 0.970 | 0.937 | 0.972 | 0.969 |
| **BIO4** | **FGOALS** | 0.947 | 0.940 | 0.954 | NA | 0.959 | 0.982 | 0.962 | 0.969 | 0.971 |
| **BIO4** | **GISS** | 0.882 | 0.907 | 0.920 | 0.959 | NA | 0.950 | 0.911 | 0.951 | 0.941 |
| **BIO4** | **IPSL** | 0.946 | 0.935 | 0.970 | 0.982 | 0.950 | NA | 0.959 | 0.980 | 0.978 |
| **BIO4** | **MIROC** | 0.952 | 0.965 | 0.937 | 0.962 | 0.911 | 0.959 | NA | 0.932 | 0.961 |
| **BIO4** | **MPI** | 0.936 | 0.914 | 0.972 | 0.969 | 0.951 | 0.980 | 0.932 | NA | 0.965 |
| **BIO4** | **MRI** | 0.943 | 0.942 | 0.969 | 0.971 | 0.941 | 0.978 | 0.961 | 0.965 | NA |
| **BIO5** | **CCSM7** | NA | 0.936 | 0.957 | 0.941 | 0.895 | 0.949 | 0.963 | 0.925 | 0.952 |
| **BIO5** | **CNRM** | 0.936 | NA | 0.934 | 0.912 | 0.882 | 0.937 | 0.933 | 0.913 | 0.959 |
| **BIO5** | **COSMOS** | 0.957 | 0.934 | NA | 0.966 | 0.929 | 0.986 | 0.975 | 0.980 | 0.980 |
| **BIO5** | **FGOALS** | 0.941 | 0.912 | 0.966 | NA | 0.968 | 0.968 | 0.982 | 0.980 | 0.972 |
| **BIO5** | **GISS** | 0.895 | 0.882 | 0.929 | 0.968 | NA | 0.933 | 0.944 | 0.962 | 0.943 |
| **BIO5** | **IPSL** | 0.949 | 0.937 | 0.986 | 0.968 | 0.933 | NA | 0.977 | 0.984 | 0.983 |
| **BIO5** | **MIROC** | 0.963 | 0.933 | 0.975 | 0.982 | 0.944 | 0.977 | NA | 0.973 | 0.976 |
| **BIO5** | **MPI** | 0.925 | 0.913 | 0.980 | 0.980 | 0.962 | 0.984 | 0.973 | NA | 0.978 |
| **BIO5** | **MRI** | 0.952 | 0.959 | 0.980 | 0.972 | 0.943 | 0.983 | 0.976 | 0.978 | NA |
| **BIO6** | **CCSM8** | NA | 0.965 | 0.981 | 0.970 | 0.943 | 0.975 | 0.973 | 0.973 | 0.982 |
| **BIO6** | **CNRM** | 0.965 | NA | 0.973 | 0.987 | 0.976 | 0.984 | 0.990 | 0.994 | 0.985 |
| **BIO6** | **COSMOS** | 0.981 | 0.973 | NA | 0.977 | 0.958 | 0.984 | 0.985 | 0.983 | 0.987 |
| **BIO6** | **FGOALS** | 0.970 | 0.987 | 0.977 | NA | 0.987 | 0.990 | 0.990 | 0.990 | 0.990 |
| **BIO6** | **GISS** | 0.943 | 0.976 | 0.958 | 0.987 | NA | 0.974 | 0.972 | 0.982 | 0.974 |
| **BIO6** | **IPSL** | 0.975 | 0.984 | 0.984 | 0.990 | 0.974 | NA | 0.989 | 0.987 | 0.989 |
| **BIO6** | **MIROC** | 0.973 | 0.990 | 0.985 | 0.990 | 0.972 | 0.989 | NA | 0.991 | 0.991 |
| **BIO6** | **MPI** | 0.973 | 0.994 | 0.983 | 0.990 | 0.982 | 0.987 | 0.991 | NA | 0.990 |
| **BIO6** | **MRI** | 0.982 | 0.985 | 0.987 | 0.990 | 0.974 | 0.989 | 0.991 | 0.990 | NA |
| **BIO7** | **CCSM9** | NA | 0.902 | 0.936 | 0.929 | 0.872 | 0.936 | 0.938 | 0.920 | 0.940 |
| **BIO7** | **CNRM** | 0.902 | NA | 0.881 | 0.939 | 0.910 | 0.913 | 0.964 | 0.909 | 0.942 |
| **BIO7** | **COSMOS** | 0.936 | 0.881 | NA | 0.936 | 0.907 | 0.967 | 0.925 | 0.958 | 0.954 |
| **BIO7** | **FGOALS** | 0.929 | 0.939 | 0.936 | NA | 0.959 | 0.971 | 0.970 | 0.962 | 0.970 |
| **BIO7** | **GISS** | 0.872 | 0.910 | 0.907 | 0.959 | NA | 0.954 | 0.932 | 0.953 | 0.940 |
| **BIO7** | **IPSL** | 0.936 | 0.913 | 0.967 | 0.971 | 0.954 | NA | 0.953 | 0.970 | 0.969 |
| **BIO7** | **MIROC** | 0.938 | 0.964 | 0.925 | 0.970 | 0.932 | 0.953 | NA | 0.939 | 0.967 |
| **BIO7** | **MPI** | 0.920 | 0.909 | 0.958 | 0.962 | 0.953 | 0.970 | 0.939 | NA | 0.960 |
| **BIO7** | **MRI** | 0.940 | 0.942 | 0.954 | 0.970 | 0.940 | 0.969 | 0.967 | 0.960 | NA |
| **BIO8** | **CCSM10** | NA | 0.865 | 0.951 | 0.948 | 0.911 | 0.913 | 0.930 | 0.915 | 0.923 |
| **BIO8** | **CNRM** | 0.865 | NA | 0.879 | 0.872 | 0.859 | 0.879 | 0.889 | 0.886 | 0.901 |
| **BIO8** | **COSMOS** | 0.951 | 0.879 | NA | 0.945 | 0.926 | 0.947 | 0.938 | 0.950 | 0.949 |
| **BIO8** | **FGOALS** | 0.948 | 0.872 | 0.945 | NA | 0.928 | 0.928 | 0.941 | 0.921 | 0.923 |
| **BIO8** | **GISS** | 0.911 | 0.859 | 0.926 | 0.928 | NA | 0.943 | 0.903 | 0.936 | 0.927 |
| **BIO8** | **IPSL** | 0.913 | 0.879 | 0.947 | 0.928 | 0.943 | NA | 0.925 | 0.966 | 0.958 |
| **BIO8** | **MIROC** | 0.930 | 0.889 | 0.938 | 0.941 | 0.903 | 0.925 | NA | 0.931 | 0.935 |
| **BIO8** | **MPI** | 0.915 | 0.886 | 0.950 | 0.921 | 0.936 | 0.966 | 0.931 | NA | 0.965 |
| **BIO8** | **MRI** | 0.923 | 0.901 | 0.949 | 0.923 | 0.927 | 0.958 | 0.935 | 0.965 | NA |
| **BIO9** | **CCSM11** | NA | 0.942 | 0.975 | 0.962 | 0.938 | 0.956 | 0.966 | 0.964 | 0.944 |
| **BIO9** | **CNRM** | 0.942 | NA | 0.934 | 0.953 | 0.941 | 0.951 | 0.943 | 0.955 | 0.932 |
| **BIO9** | **COSMOS** | 0.975 | 0.934 | NA | 0.950 | 0.923 | 0.948 | 0.963 | 0.960 | 0.930 |
| **BIO9** | **FGOALS** | 0.962 | 0.953 | 0.950 | NA | 0.970 | 0.966 | 0.964 | 0.964 | 0.944 |
| **BIO9** | **GISS** | 0.938 | 0.941 | 0.923 | 0.970 | NA | 0.956 | 0.945 | 0.963 | 0.939 |
| **BIO9** | **IPSL** | 0.956 | 0.951 | 0.948 | 0.966 | 0.956 | NA | 0.962 | 0.967 | 0.953 |
| **BIO9** | **MIROC** | 0.966 | 0.943 | 0.963 | 0.964 | 0.945 | 0.962 | NA | 0.963 | 0.950 |
| **BIO9** | **MPI** | 0.964 | 0.955 | 0.960 | 0.964 | 0.963 | 0.967 | 0.963 | NA | 0.953 |
| **BIO9** | **MRI** | 0.944 | 0.932 | 0.930 | 0.944 | 0.939 | 0.953 | 0.950 | 0.953 | NA |
| **BIO10** | **CCSM12** | NA | 0.959 | 0.981 | 0.980 | 0.952 | 0.972 | 0.987 | 0.975 | 0.982 |
| **BIO10** | **CNRM** | 0.959 | NA | 0.958 | 0.946 | 0.927 | 0.958 | 0.958 | 0.956 | 0.978 |
| **BIO10** | **COSMOS** | 0.981 | 0.958 | NA | 0.978 | 0.953 | 0.987 | 0.984 | 0.992 | 0.987 |
| **BIO10** | **FGOALS** | 0.980 | 0.946 | 0.978 | NA | 0.975 | 0.982 | 0.989 | 0.984 | 0.979 |
| **BIO10** | **GISS** | 0.952 | 0.927 | 0.953 | 0.975 | NA | 0.960 | 0.964 | 0.965 | 0.960 |
| **BIO10** | **IPSL** | 0.972 | 0.958 | 0.987 | 0.982 | 0.960 | NA | 0.984 | 0.994 | 0.986 |
| **BIO10** | **MIROC** | 0.987 | 0.958 | 0.984 | 0.989 | 0.964 | 0.984 | NA | 0.984 | 0.983 |
| **BIO10** | **MPI** | 0.975 | 0.956 | 0.992 | 0.984 | 0.965 | 0.994 | 0.984 | NA | 0.987 |
| **BIO10** | **MRI** | 0.982 | 0.978 | 0.987 | 0.979 | 0.960 | 0.986 | 0.983 | 0.987 | NA |
| **BIO11** | **CCSM13** | NA | 0.976 | 0.987 | 0.976 | 0.955 | 0.979 | 0.984 | 0.982 | 0.986 |
| **BIO11** | **CNRM** | 0.976 | NA | 0.986 | 0.988 | 0.980 | 0.995 | 0.991 | 0.996 | 0.989 |
| **BIO11** | **COSMOS** | 0.987 | 0.986 | NA | 0.981 | 0.965 | 0.989 | 0.993 | 0.991 | 0.990 |
| **BIO11** | **FGOALS** | 0.976 | 0.988 | 0.981 | NA | 0.988 | 0.989 | 0.990 | 0.990 | 0.992 |
| **BIO11** | **GISS** | 0.955 | 0.980 | 0.965 | 0.988 | NA | 0.978 | 0.974 | 0.981 | 0.978 |
| **BIO11** | **IPSL** | 0.979 | 0.995 | 0.989 | 0.989 | 0.978 | NA | 0.993 | 0.995 | 0.991 |
| **BIO11** | **MIROC** | 0.984 | 0.991 | 0.993 | 0.990 | 0.974 | 0.993 | NA | 0.991 | 0.993 |
| **BIO11** | **MPI** | 0.982 | 0.996 | 0.991 | 0.990 | 0.981 | 0.995 | 0.991 | NA | 0.991 |
| **BIO11** | **MRI** | 0.986 | 0.989 | 0.990 | 0.992 | 0.978 | 0.991 | 0.993 | 0.991 | NA |
| **BIO12** | **CCSM14** | NA | 0.896 | 0.767 | 0.872 | 0.850 | 0.887 | 0.890 | 0.841 | 0.842 |
| **BIO12** | **CNRM** | 0.896 | NA | 0.742 | 0.897 | 0.876 | 0.906 | 0.878 | 0.870 | 0.894 |
| **BIO12** | **COSMOS** | 0.767 | 0.742 | NA | 0.629 | 0.700 | 0.721 | 0.736 | 0.890 | 0.743 |
| **BIO12** | **FGOALS** | 0.872 | 0.897 | 0.629 | NA | 0.864 | 0.865 | 0.840 | 0.752 | 0.813 |
| **BIO12** | **GISS** | 0.850 | 0.876 | 0.700 | 0.864 | NA | 0.841 | 0.821 | 0.767 | 0.834 |
| **BIO12** | **IPSL** | 0.887 | 0.906 | 0.721 | 0.865 | 0.841 | NA | 0.861 | 0.849 | 0.871 |
| **BIO12** | **MIROC** | 0.890 | 0.878 | 0.736 | 0.840 | 0.821 | 0.861 | NA | 0.836 | 0.774 |
| **BIO12** | **MPI** | 0.841 | 0.870 | 0.890 | 0.752 | 0.767 | 0.849 | 0.836 | NA | 0.857 |
| **BIO12** | **MRI** | 0.842 | 0.894 | 0.743 | 0.813 | 0.834 | 0.871 | 0.774 | 0.857 | NA |
| **BIO13** | **CCSM15** | NA | 0.866 | 0.754 | 0.863 | 0.843 | 0.885 | 0.875 | 0.845 | 0.838 |
| **BIO13** | **CNRM** | 0.866 | NA | 0.776 | 0.842 | 0.859 | 0.874 | 0.860 | 0.878 | 0.863 |
| **BIO13** | **COSMOS** | 0.754 | 0.776 | NA | 0.687 | 0.725 | 0.750 | 0.732 | 0.895 | 0.768 |
| **BIO13** | **FGOALS** | 0.863 | 0.842 | 0.687 | NA | 0.849 | 0.838 | 0.838 | 0.789 | 0.810 |
| **BIO13** | **GISS** | 0.843 | 0.859 | 0.725 | 0.849 | NA | 0.849 | 0.823 | 0.791 | 0.837 |
| **BIO13** | **IPSL** | 0.885 | 0.874 | 0.750 | 0.838 | 0.849 | NA | 0.853 | 0.852 | 0.854 |
| **BIO13** | **MIROC** | 0.875 | 0.860 | 0.732 | 0.838 | 0.823 | 0.853 | NA | 0.828 | 0.766 |
| **BIO13** | **MPI** | 0.845 | 0.878 | 0.895 | 0.789 | 0.791 | 0.852 | 0.828 | NA | 0.857 |
| **BIO13** | **MRI** | 0.838 | 0.863 | 0.768 | 0.810 | 0.837 | 0.854 | 0.766 | 0.857 | NA |
| **BIO14** | **CCSM16** | NA | 0.745 | 0.602 | 0.750 | 0.707 | 0.740 | 0.771 | 0.658 | 0.672 |
| **BIO14** | **CNRM** | 0.745 | NA | 0.614 | 0.838 | 0.821 | 0.871 | 0.805 | 0.811 | 0.808 |
| **BIO14** | **COSMOS** | 0.602 | 0.614 | NA | 0.485 | 0.553 | 0.594 | 0.669 | 0.783 | 0.524 |
| **BIO14** | **FGOALS** | 0.750 | 0.838 | 0.485 | NA | 0.809 | 0.794 | 0.724 | 0.649 | 0.710 |
| **BIO14** | **GISS** | 0.707 | 0.821 | 0.553 | 0.809 | NA | 0.764 | 0.719 | 0.664 | 0.714 |
| **BIO14** | **IPSL** | 0.740 | 0.871 | 0.594 | 0.794 | 0.764 | NA | 0.775 | 0.790 | 0.787 |
| **BIO14** | **MIROC** | 0.771 | 0.805 | 0.669 | 0.724 | 0.719 | 0.775 | NA | 0.800 | 0.647 |
| **BIO14** | **MPI** | 0.658 | 0.811 | 0.783 | 0.649 | 0.664 | 0.790 | 0.800 | NA | 0.725 |
| **BIO14** | **MRI** | 0.672 | 0.808 | 0.524 | 0.710 | 0.714 | 0.787 | 0.647 | 0.725 | NA |
| **BIO15** | **CCSM17** | NA | 0.559 | 0.433 | 0.689 | 0.649 | 0.715 | 0.755 | 0.725 | 0.700 |
| **BIO15** | **CNRM** | 0.559 | NA | 0.312 | 0.638 | 0.652 | 0.564 | 0.633 | 0.628 | 0.663 |
| **BIO15** | **COSMOS** | 0.433 | 0.312 | NA | 0.319 | 0.337 | 0.378 | 0.386 | 0.466 | 0.377 |
| **BIO15** | **FGOALS** | 0.689 | 0.638 | 0.319 | NA | 0.770 | 0.692 | 0.725 | 0.672 | 0.733 |
| **BIO15** | **GISS** | 0.649 | 0.652 | 0.337 | 0.770 | NA | 0.751 | 0.709 | 0.718 | 0.736 |
| **BIO15** | **IPSL** | 0.715 | 0.564 | 0.378 | 0.692 | 0.751 | NA | 0.705 | 0.717 | 0.732 |
| **BIO15** | **MIROC** | 0.755 | 0.633 | 0.386 | 0.725 | 0.709 | 0.705 | NA | 0.770 | 0.730 |
| **BIO15** | **MPI** | 0.725 | 0.628 | 0.466 | 0.672 | 0.718 | 0.717 | 0.770 | NA | 0.805 |
| **BIO15** | **MRI** | 0.700 | 0.663 | 0.377 | 0.733 | 0.736 | 0.732 | 0.730 | 0.805 | NA |
| **BIO16** | **CCSM18** | NA | 0.863 | 0.759 | 0.856 | 0.838 | 0.878 | 0.876 | 0.848 | 0.832 |
| **BIO16** | **CNRM** | 0.863 | NA | 0.761 | 0.847 | 0.852 | 0.876 | 0.855 | 0.872 | 0.862 |
| **BIO16** | **COSMOS** | 0.759 | 0.761 | NA | 0.662 | 0.710 | 0.732 | 0.722 | 0.891 | 0.756 |
| **BIO16** | **FGOALS** | 0.856 | 0.847 | 0.662 | NA | 0.844 | 0.841 | 0.840 | 0.773 | 0.802 |
| **BIO16** | **GISS** | 0.838 | 0.852 | 0.710 | 0.844 | NA | 0.842 | 0.812 | 0.776 | 0.829 |
| **BIO16** | **IPSL** | 0.878 | 0.876 | 0.732 | 0.841 | 0.842 | NA | 0.843 | 0.845 | 0.856 |
| **BIO16** | **MIROC** | 0.876 | 0.855 | 0.722 | 0.840 | 0.812 | 0.843 | NA | 0.821 | 0.754 |
| **BIO16** | **MPI** | 0.848 | 0.872 | 0.891 | 0.773 | 0.776 | 0.845 | 0.821 | NA | 0.853 |
| **BIO16** | **MRI** | 0.832 | 0.862 | 0.756 | 0.802 | 0.829 | 0.856 | 0.754 | 0.853 | NA |
| **BIO17** | **CCSM19** | NA | 0.775 | 0.637 | 0.768 | 0.742 | 0.759 | 0.785 | 0.688 | 0.690 |
| **BIO17** | **CNRM** | 0.775 | NA | 0.642 | 0.855 | 0.846 | 0.884 | 0.820 | 0.821 | 0.837 |
| **BIO17** | **COSMOS** | 0.637 | 0.642 | NA | 0.490 | 0.590 | 0.601 | 0.672 | 0.809 | 0.551 |
| **BIO17** | **FGOALS** | 0.768 | 0.855 | 0.490 | NA | 0.833 | 0.811 | 0.745 | 0.661 | 0.737 |
| **BIO17** | **GISS** | 0.742 | 0.846 | 0.590 | 0.833 | NA | 0.791 | 0.748 | 0.693 | 0.745 |
| **BIO17** | **IPSL** | 0.759 | 0.884 | 0.601 | 0.811 | 0.791 | NA | 0.808 | 0.796 | 0.820 |
| **BIO17** | **MIROC** | 0.785 | 0.820 | 0.672 | 0.745 | 0.748 | 0.808 | NA | 0.800 | 0.687 |
| **BIO17** | **MPI** | 0.688 | 0.821 | 0.809 | 0.661 | 0.693 | 0.796 | 0.800 | NA | 0.758 |
| **BIO17** | **MRI** | 0.690 | 0.837 | 0.551 | 0.737 | 0.745 | 0.820 | 0.687 | 0.758 | NA |
| **BIO18** | **CCSM20** | NA | 0.812 | 0.684 | 0.820 | 0.793 | 0.840 | 0.819 | 0.776 | 0.733 |
| **BIO18** | **CNRM** | 0.812 | NA | 0.734 | 0.798 | 0.801 | 0.811 | 0.791 | 0.828 | 0.816 |
| **BIO18** | **COSMOS** | 0.684 | 0.734 | NA | 0.622 | 0.648 | 0.690 | 0.652 | 0.860 | 0.724 |
| **BIO18** | **FGOALS** | 0.820 | 0.798 | 0.622 | NA | 0.784 | 0.797 | 0.778 | 0.721 | 0.724 |
| **BIO18** | **GISS** | 0.793 | 0.801 | 0.648 | 0.784 | NA | 0.802 | 0.753 | 0.725 | 0.756 |
| **BIO18** | **IPSL** | 0.840 | 0.811 | 0.690 | 0.797 | 0.802 | NA | 0.790 | 0.799 | 0.770 |
| **BIO18** | **MIROC** | 0.819 | 0.791 | 0.652 | 0.778 | 0.753 | 0.790 | NA | 0.760 | 0.646 |
| **BIO18** | **MPI** | 0.776 | 0.828 | 0.860 | 0.721 | 0.725 | 0.799 | 0.760 | NA | 0.797 |
| **BIO18** | **MRI** | 0.733 | 0.816 | 0.724 | 0.724 | 0.756 | 0.770 | 0.646 | 0.797 | NA |
| **BIO19** | **CCSM21** | NA | 0.704 | 0.613 | 0.765 | 0.742 | 0.697 | 0.710 | 0.674 | 0.677 |
| **BIO19** | **CNRM** | 0.704 | NA | 0.590 | 0.760 | 0.790 | 0.830 | 0.782 | 0.760 | 0.796 |
| **BIO19** | **COSMOS** | 0.613 | 0.590 | NA | 0.477 | 0.612 | 0.591 | 0.654 | 0.774 | 0.568 |
| **BIO19** | **FGOALS** | 0.765 | 0.760 | 0.477 | NA | 0.780 | 0.724 | 0.690 | 0.636 | 0.679 |
| **BIO19** | **GISS** | 0.742 | 0.790 | 0.612 | 0.780 | NA | 0.734 | 0.731 | 0.685 | 0.748 |
| **BIO19** | **IPSL** | 0.697 | 0.830 | 0.591 | 0.724 | 0.734 | NA | 0.748 | 0.758 | 0.787 |
| **BIO19** | **MIROC** | 0.710 | 0.782 | 0.654 | 0.690 | 0.731 | 0.748 | NA | 0.753 | 0.705 |
| **BIO19** | **MPI** | 0.674 | 0.760 | 0.774 | 0.636 | 0.685 | 0.758 | 0.753 | NA | 0.716 |
| **BIO19** | **MRI** | 0.677 | 0.796 | 0.568 | 0.679 | 0.748 | 0.787 | 0.705 | 0.716 | NA |
